# Assessment of task-based image quality for abdominal CT protocols linked with national diagnostic reference levels

**DOI:** 10.1007/s00330-021-08185-1

**Published:** 2021-07-29

**Authors:** Anaïs Viry, Christoph Aberle, Thiago Lima, Reto Treier, Sebastian T. Schindera, Francis R. Verdun, Damien Racine

**Affiliations:** 1 Institute of Radiation Physics, Lausanne University Hospital and University of Lausanne, Lausanne, Switzerland; 2 Department of Radiology, University Hospital Basel, University of Basel, Basel, Switzerland; 3 Department of Radiology and Nuclear Medicine, Kantonsspital Luzern, Lucerne, Switzerland; 4 Radiological Protection Division, Federal Office of Public Health, Bern, Switzerland; 5 Department of Radiology, Kantonsspital Aarau, Aarau, Switzerland

**Keywords:** Multidetector computed tomography, Diagnostic reference levels, Radiation protection

## Abstract

**Objectives:**

To assess task-based image quality for two abdominal protocols on various CT scanners. To establish a relationship between diagnostic reference levels (DRLs) and task-based image quality.

**Methods:**

A protocol for the detection of focal liver lesions was used to scan an anthropomorphic abdominal phantom containing 8- and 5-mm low-contrast (20 HU) spheres at five CTDI_vol_ levels (4, 8, 12, 16, and 20 mGy) on 12 CTs. Another phantom with high-contrast calcium targets (200 HU) was scanned at 2, 4, 6, 10, and 15 mGy using a renal stones protocol on the same CTs. To assess the detectability, a channelized Hotelling observer was used for low-contrast targets and a non-prewhitening observer with an eye filter was used for high contrast targets. The area under the ROC curve and signal to noise ratio were used as figures of merit.

**Results:**

For the detection of 8-mm spheres, the image quality reached a high level (mean AUC over all CTs higher than 0.95) at 11 mGy. For the detection of 5-mm spheres, the AUC never reached a high level of image quality. Variability between CTs was found, especially at low dose levels. For the search of renal stones, the AUC was nearly maximal even for the lowest dose level.

**Conclusions:**

Comparable task-based image quality cannot be reached at the same dose level on all CT scanners. This variability implies the need for scanner-specific dose optimization.

**Key Points:**

*• There is an image quality variability for subtle low-contrast lesion detection in the clinically used dose range.*

*• Diagnostic reference levels were linked with task-based image quality metrics.*

*• There is a need for specific dose optimization for each CT scanner and clinical protocol.*

## Introduction

The contribution of computed tomography (CT) to the total effective dose due to medical X-ray examinations has been recently reported to be up to 70% [1]. Hence, continuous efforts have been made by manufacturers and users of CT to reduce the dose level per examination with the integration of new technologies (e.g., tube current modulation, iterative reconstruction (IR) algorithms, or more efficient detectors) and the optimization of clinical protocols [2].

An important aspect to take into account when dealing with protocol optimization is the variation of the practice even for a well-defined indication. Hence, diagnostic reference levels (DRLs) were proposed by the International Commission on Radiological Protection (ICRP) in 1996 to reduce the variability of clinical practice by leading users of CT to take actions when the local dose indicator systematically exceeds the national DRL [3]. Two major limitations appear. DRLs are often not related to precise clinical indication, nor to any clinical image quality criteria. The first limitation was partially addressed by recently published national or local DRLs [4–9], and at the European level [10]; the second one is still an open question as mentioned by Rehani [11]. Moreover, the technological differences between CT scanners should be taken into account when dealing with clinical protocol optimization. Adjusting the radiation dose level of a clinical protocol using the value of the associated DRL without assessing the image quality is suboptimal [12]. On the one hand, further patient dose optimization could be justified for the most modern CT scanners. On the other hand, it could cause an excessive dose reduction with a loss of diagnostic performance, in particular for older CT scanners. This practice can lead to variations in image quality and patient care, while the goal is the standardization of image quality such that it is just sufficient for the clinical task at the lowest possible dose [13, 14]. Hence, it appears necessary to associate national DRLs for specific clinical tasks with task-based image quality criteria in order to assess a potential dose optimization and avoid excessive patient dose reduction.

Among all existing CT examinations, abdominal CT protocols deliver the highest radiation doses to the patients [1]. Moreover, the optimization process is particularly crucial for abdominal protocols due to the challenges arising from the detection of small low-contrast lesions [15]. An excessive patient dose reduction can highly increase the risk of missing subtle lesions.

The use of basic image quality metrics (standard deviation, contrast, contrast-to-noise ratio, modulation transfer function) is of limited interest because they are not directly related to any clinical requirement [16]. Task-based image quality analysis was initially proposed by Barrett and Myers to quantify the CT diagnostic performances [17, 18]. The methodology was recently applied with success to benchmark CT scanners [19] and clinical protocols [20] or assess the use of IR algorithms [16, 21].

The purpose of this contribution is to assess task-based image quality for two abdominal protocols on various CT scanners and to establish a relationship between DRL values and image quality for the respective clinical tasks.

## Materials and methods

### Image quality phantoms

**Fig. 1:**
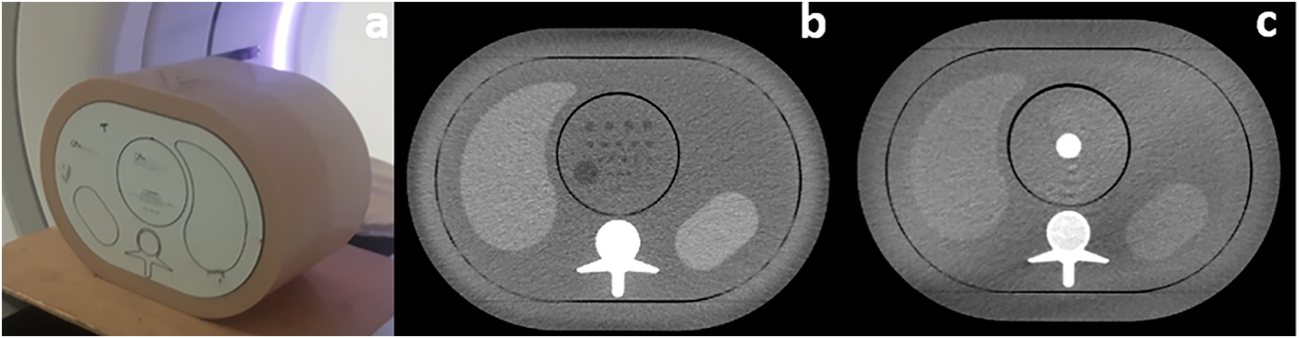
**a** Photo of the QRM phantom. **b** CT slice of the phantom with the module containing the low-contrast spheres, the 8-mm spheres are all positioned on the first row and the 5-mm spheres are positioned on the third row. **c** CT slice of the phantom with the module containing the high contrast rod

An abdominal anthropomorphic phantom (QRM, A PTW COMPANY) was used to assess the image quality of two examination types. The phantom mimics various tissues (muscle, liver, spleen, and vertebrae) (Fig. 1a). Due to the absence of materials with high atomic numbers, the phantom was designed to assess non-contrast CT scans. Its effective diameter of 30 cm simulates the attenuation of a patient with a weight around 75 kg. The phantom contains a hole of 10 cm in diameter into which different modules can be inserted. To mimic the detection of focal liver lesions, a first module containing hypodense low-contrast spheres of different sizes (in particular 8 and 5 mm diameter) with a contrast of 20 HU relative to the background was used (Fig. 1b). These two lesion sizes were considered clinically relevant. Indeed, liver lesions smaller than 5 mm are often benign. Furthermore, it is difficult to accurately characterize smaller lesion sizes in the liver with this type of contrast in CT [22].

A second module containing a high contrast calcic rod of 20 mm in diameter and a contrast of 200 HU was used to quantify the spatial resolution, an important aspect for assessing the detection of renal stones (Fig. 1c).

### CT scanners and acquisition/reconstruction parameters

In concertation with a panel of radiologists, two sets of acquisition and reconstruction parameter settings were defined that are typical for examinations of a) focal liver lesions and b) renal stones. Five volume computed tomography dose index (CTDI_vol_) levels were used for each set (4, 8, 12, 16, and 20 mGy for focal liver lesions and 2, 4, 6, 10, and 15 mGy for renal stones). The current Swiss DRLs (11 mGy for focal liver lesions CT acquisitions and 6 mGy for renal stones CT acquisitions) and the underlying dose distributions [6] were used to determine the 5 CTDI_vol_ levels, so that they cover the clinically relevant dose range.

The 12 CT scanners involved in this study are listed in Table 1. Three different CT scanners from each of the four major CT manufacturers were included. Thus, the variability of image quality due to scanner-specific technology properties could be adequately studied. In practice, there is no identical set of acquisition and reconstruction parameters that can be used on all CT scanner models. Instead, acquisition and reconstruction parameters were matched as closely as possible (Table 1). Reconstruction algorithms and reconstruction kernels are manufacturer- and model-specific.

**Table 1:** CT scanners and their acquisition and reconstruction settings

Manufacturer and model	Tube voltage [kV]	ATCM	Pitch ^b^	Rotation time [s] ^d^	Collimation [mm]	Slice thickness / increment ^f^	Reconstruction algorithm/kernel
Philips Brilliance 16	120	x-y	0.938	0.75	24	3 mm/1.5 mm	FBP / Standard (B)
Philips Ingenuity CT	120	x-y-z	0.999 (RS); 1.404 (FLL)	0.75 (RS); 0.75 (FLL, 4, 8, 12, 16 mGy); 1.0 (FLL, 20 mGy)^e^	40	2.5 mm/1.25 mm	iDose^4^ level 3 (out of 6) / Standard (B)
Philips Brilliance iCT 256	120	z	1.014 (RS); 1.142 (FLL)	0.75	40	2.5 mm/1.25 mm	iDose^4^ level 3 (out of 6) / Standard (B)
Canon Aquilion RXL	120	OFF ^a^ (RS); x-y-z (FLL)	1.000 (RS); 1.313 (FLL)	0.75	32	3 mm/1.5 mm	AIDR 3D Standard / FC18
Canon Aquilion CXL	120	OFF ^a^	0.906 (RS); 1.297 (FLL)	0.75	32	3 mm/1.5 mm	AIDR 3D Standard / FC18
Canon Aquilion ONE ViSION Edition	120	x-y-z	0.950 (RS); 1.300 (FLL)	0.75	40	3 mm/1.5 mm	AIDR 3D-enhanced Standard / FC08
GE BrightSpeed S	120	x-y-z	0.938 (RS, 2, 6, 10, 15 mGy); 1.375 (RS, 4 mGy)^c^; 1.375 (FLL)	0.8 (RS); 0.8 (FLL, 4, 8, 12, 16 mGy); 1.0 (FLL, 20 mGy)^e^	20	2.5 mm/1.25 mm	ASIR 50% / Soft
GE Discovery CT750 HD	120	x-y-z	0.984 (RS); 1.375 (FLL)	0.8	40	2.5 mm/1.25 mm	ASIR-V 70% / Standard (RS) ASIR-V 50% / Standard (FLL)
GE Revolution CT	120	x-y-z	0.984 (RS); 1.375 (FLL)	0.8	40	2.5 mm/1.25 mm	ASIR-V 50% / Standard
Siemens SOMATOM Emotion 16	130	x-y-z	1.000 (RS); 1.300 (FLL)	1.0 (RS, 4, 6, 10, 15 mGy); 0.6 (RS, 2 mGy)^e^; 1.0 (FLL)	19.2	3 mm/1.5 mm	FBP / B30s
Siemens SOMATOM Definition Edge	120	x-y-z	1.000 (RS); 1.300 (FLL)	1.0	38.4	3 mm/1.5 mm	ADMIRE strength 3 (out of 5) / I30s
Siemens SOMATOM Force	120	x-y-z	1.000 (RS); 1.300 (FLL)	1.0	57.6	3 mm/1.5 mm	ADMIRE strength 3 (out of 5) / Bf40s

^a^Automatic tube current modulation (ATCM) led to very strong, unstable modulation due to the sharp edge of the phantom and had to be turned off

^b^Pitch: as close as possible to 1.0 (renal stones, RS) and 1.3 (focal liver lesions, FLL)

^c^Tube current limitations required a pitch adjustment

^d^Rotation time: as close as possible to 0.8 s

^e^Tube current limitations required a rotation time adjustment

^f^The displayed field of view (FOV) was always 370 mm

### Radiation dose assessment

Before each acquisition session, CTDI_w_ was measured with a 10-cm ionization chamber (PTW TM30009 or Radcal 10X6-3CT) using a 32-cm-diameter CTDI phantom, following the international electrotechnical commission (IEC) standard 60601-2-44. The ratio of the measured CTDI_w_ to the displayed CTDI_w_ was used to correct the displayed CTDI_vol_ of the image quality phantom scans. For the 12 CT scanners, the correction factors ranged from 0.847 to 1.057. Furthermore, the actual radiation dose depends on the z-position if the tube current is modulated. All CTDI_vol_ values presented in the results section are corrected and refer to the actual z-position where the image quality was evaluated.

Relative standard uncertainties on the final CTDI_vol_ values were evaluated in detail [23]. It turned out that 2.5% is a good estimate for all CT scanners and all dose levels. The most important uncertainty component was the uncertainty of the CTDI_w_ measurements, more specifically the uncertainty of the chamber calibration factors (relative standard uncertainty of 1.5%, from calibration certificate).

### Image analysis

#### Low-contrast detectability

We quantitatively assessed the image quality using a task-based methodology. The clinical tasks were the detection of low contrast lesions with a size of 5 and 8 mm. The low-contrast module contains four spheres of 8 mm and five spheres of 5 mm in diameter in the exact same slice. As 20 acquisitions for each dose level were acquired, we were able to extract at least 80 square regions of interest (ROIs) of 18 × 18 pixels containing lesions of 8 mm and 5 mm in diameter. On the right homogeneous part of the phantom images, 400 ROIs containing only noise were extracted in five slices around the slice of interest (Fig. 1b).

An anthropomorphic mathematical model observer was chosen to quantitatively assess the detectability of low contrast lesions. Based on Bayesian statistical decision theory, this kind of observer has the ability to mimic human observer responses in the detection of low contrast structures in an image [24–26]. The channelized Hotelling observer (CHO) with 10 dense difference of Gaussian channels (DDoG) was applied, following the methodology proposed by Wunderlich et al to compute the signal-to-noise ratio (SNR), expressing the detectability of the lesion [27]. The CHO model observer was previously computed using the same anthropomorphic phantom [21]. As CHO model observers are more efficient than human observers for simple detection tasks in uniform background, it is necessary to adjust the detection outcomes of model observers by adding internal noise on the covariance matrix [28]. Internal noise was calibrated with the data from the inter-comparison study of Ba et al [29]. The area under the receiver operating characteristics curve (AUC) was used as the figure of merit to assess the detectability of low contrast lesions. A monotonic function can link SNR and AUC [30]. The AUC was computed for each CT, dose level, and lesion size.

#### High-contrast detectability

For the detection of renal stones, we also used a task-based methodology. The clinical task was the detection of calcic lesions of 3 and 5 mm with a contrast of 450 HU. Indeed, renal stones of 3 mm and smaller have a high chance of spontaneous passage [31]. We decided to use 3 mm as a cut-off. An anthropomorphic mathematical observer, the non-prewhitening observer with an eye filter (NPWE) expressed in the Fourier domain was used. Developed by Burgess [32], the NPWE computes the SNR of simulated high contrast lesions using the in-plane contrast-dependent spatial resolution (target transfer function (TTF)) from the images of high contrast objects, the noise power spectrum (NPS), and the virtual transfer function of the human eye [33].

The TTF was computed using the module containing the high-contrast rod. As six acquisitions were performed for each CT scanner and dose level, 78 ROIs of 64 × 64 pixels centered on the rod could be extracted. The 2D TTF was calculated from the edge of the rod following the methodology described by Monnin et al and radially averaged and normalized at the zero frequency to obtain the 1D TTF [34].

Image noise was quantified by computing the NPS [35–37]. A total of 90 ROIs of 64 × 64 pixels were extracted from 15 homogeneous slices per acquisition. The 2D NPS was computed on the cropped ROIs and then radially averaged to obtain 1D NPS.

As the integral of 1D NPS decreases as the slice thickness increases [38], we corrected the SNR of the NPWE model for the 5 CT scanners with a 2.5 mm slice thickness by a factor $\sqrt{\frac{3}{2.5}}$.

### Statistical analysis

For the CHO model observer, to reduce the positive bias caused by the use of a finite number of images and to compute the exact 95% confidence interval of SNR, the methodology developed by Wunderlich was applied [27]. A linear fit between the logarithm of the SNR and the logarithm of the dose, taking into account the uncertainties, was performed for each CT scanner to calculate SNR and AUC values at a given CTDI_vol_ and vice versa.

For the NPWE outcome, the uncertainties were determined using a bootstrap method. Results were computed using 100 bootstrapped samples of 50 ROIs used for TTF and NPS calculations.

## Results

**Fig. 2:**
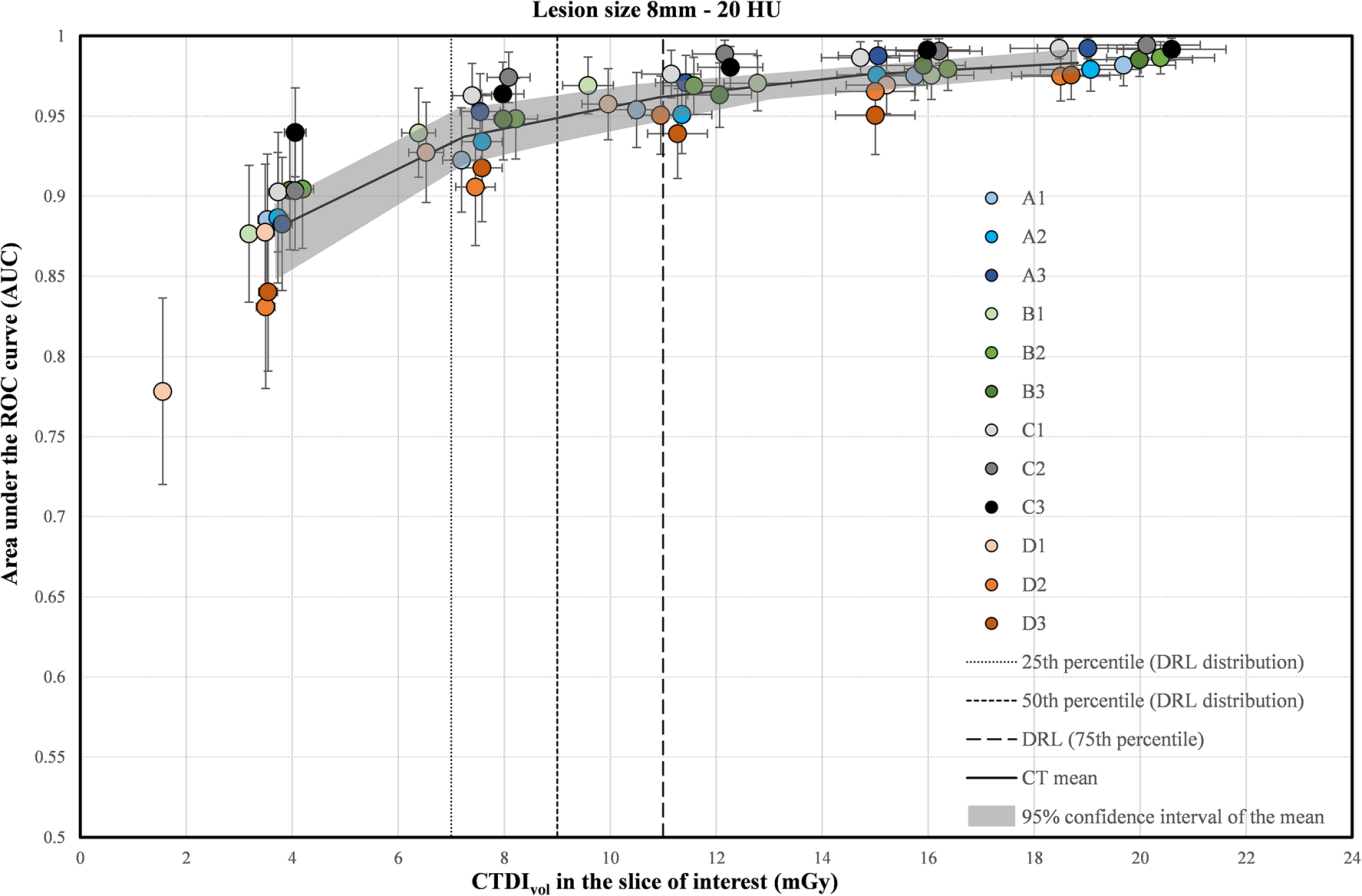
Area under the ROC curve as a function of CTDI_vol_ in the slice of interest for the 8-mm lesion size for the 12 CT scanners. The horizontal and vertical uncertainty bars represent the expanded uncertainty (k = 2, 95% level of confidence) for the CTDI_vol_ and AUC, respectively. The solid black line was plotted by joining 5 points representing the mean AUC and the mean CTDI_vol_ over all 12 CTs for each dose level. The gray band was plotted by joining the limits of the 95% confidence intervals of the 5 points

**Fig. 3:**
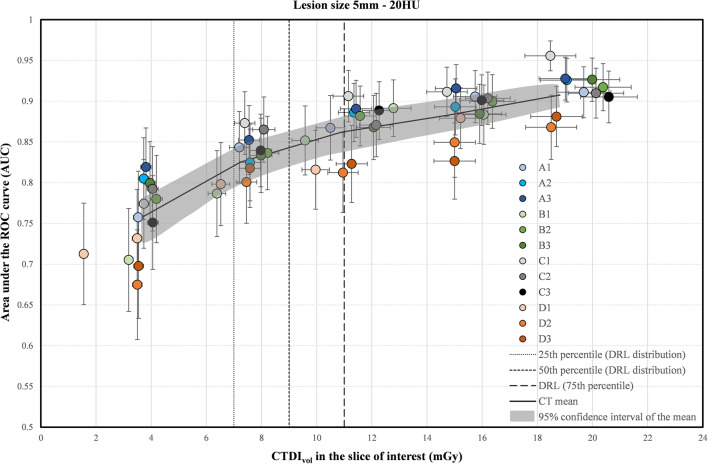
Area under the ROC curve as a function of CTDI_vol_ in the slice of interest for the 5-mm lesion size for the 12 CT scanners. The horizontal and vertical uncertainty bars represent the expanded uncertainty (k = 2, 95% level of confidence) for the CTDI_vol_ and AUC, respectively. The solid black line was plotted by joining 5 points representing the mean AUC and the mean CTDI_vol_ over all 12 CTs for each dose level. The gray band was plotted by joining the limits of the 95% confidence intervals of the 5 points

To ensure the impartiality of this work, the results are reported in an anonymous manner consistently throughout the manuscript. It was not the purpose of this work to compare individual CT scanner models but rather to study the size of the variability when using different models. A capital letter (A, B, C, and D) was assigned to each manufacturer and figures 1, 2, and 3 refer to the three different CT scanners.

### Low-contrast detectability

As expected, irrespective of the lesion size, the low contrast detectability increased with the dose level (Figs. 2 and 3).

For the largest lesion size (8 mm), at 11 mGy, corresponding to the Swiss DRL of the investigated liver protocol, the AUC reached a high image quality level with values higher than 0.95 for 10 out of 12 CT scanners (Fig. 2 and Table 2). The use of a dose level below 7 mGy (25^th^ percentile of the DRL distribution) induced a loss of image quality. The percentage of AUC reduction when decreasing the dose level from 11 to 7 mGy, varied from 1.7 to 4.3% for the various CT scanners. The variability of image quality between the various CTs is higher at low-dose levels: The AUC ranged from 0.90 to 0.96 at 7 mGy (Table 2). A comparable level of image quality was obtained at substantially different CTDI_vol_ values. For example, an AUC of 0.95 was obtained at a range of doses between 5.3 and 13 mGy, as calculated using the best-fit equations.

**Table 2:** AUC values for the 8 mm (top) and 5 mm (bottom) lesion size calculated using the fit equation for each CT scanner at three dose levels: the 25th percentile of the DRL distribution, the 50th percentile (achievable dose), and the 75th percentile (DRL). The percentage of AUC reduction was then calculated when decreasing the dose level from the 75th percentile to the 25th percentile of the DRL distribution

**CT scanner**	**AUC (11 mGy, 8 mm lesions)**	**95% confidence interval**	**AUC (9 mGy, 8 mm lesions)**	**95% confidence interval**	**AUC (7 mGy, 8 mm lesions)**	**95% confidence interval**	**AUC reduction (11 to 7 mGy)**
A1	0.956	[0.947–0.965]	0.945	[0.930–0.959]	0.929	[0.914–0.944]	2.8%
A2	0.956	[0.947–0.965]	0.944	[0.929–0.959]	0.928	[0.913–0.944]	2.8%
A3	0.972	[0.966–0.978]	0.960	[0.945–0.974]	0.941	[0.927–0.956]	3.1%
B1	0.965	[0.957–0.973]	0.955	[0.942–0.967]	0.940	[0.927–0.952]	2.6%
B2	0.964	[0.956–0.971]	0.954	[0.939–0.968]	0.939	[0.924–0.953]	2.6%
B3	0.964	[0.957–0.971]	0.954	[0.939–0.967]	0.939	[0.925–0.954]	2.5%
C1	0.977	[0.971–0.982]	0.967	[0.955–0.978]	0.952	[0.940–0.964]	2.4%
C2	0.980	[0.975–0.985]	0.971	[0.959–0.982]	0.956	[0.944–0.969]	2.4%
C3	0.979	[0.973–0.983]	0.972	[0.965–0.978]	0.963	[0.952–0.972]	1.7%
D1	0.959	[0.948–0.970]	0.946	[0.933–0.958]	0.926	[0.913–0.939]	3.3%
D2	0.943	[0.932–0.954]	0.926	[0.906–0.946]	0.903	[0.884–0.922]	4.3%
D3	0.938	[0.927–0.950]	0.924	[0.904–0.943]	0.903	[0.884–0.922]	3.8%
**CT scanner**	**AUC (11 mGy, 5 mm lesions)**	**95% confidence interval**	**AUC (9 mGy, 5 mm lesions)**	**95% confidence interval**	**AUC (7 mGy, 5 mm lesions)**	**95% confidence interval**	**AUC reduction (11 to 7 mGy)**
A1	0.872	[0.853–0.891]	0.854	[0.827–0.880]	0.830	[0.801–0.858]	4.8%
A2	0.893	[0.877–0.909]	0.864	[0.839–0.888]	0.845	[0.819–0.871]	4.0%
A3	0.879	[0.861–0.897]	0.879	[0.855–0.902]	0.860	[0.837–0.884]	3.6%
B1	0.839	[0.816–0.862]	0.814	[0.782–0.844]	0.783	[0.752–0.813]	6.7%
B2	0.870	[0.852–0.889]	0.852	[0.822–0.880]	0.829	[0.799–0.859]	4.7%
B3	0.877	[0.859–0.895]	0.861	[0.834–0.887]	0.840	[0.813–0.868]	4.2%
C1	0.902	[0.886–0.918]	0.879	[0.853–0.905]	0.851	[0.825–0.876]	5.7%
C2	0.876	[0.858–0.893]	0.861	[0.834–0.887]	0.842	[0.815–0.868]	3.9%
C3	0.864	[0.844–0.884]	0.844	[0.819–0.869]	0.819	[0.787–0.849]	5.6%
D1	0.838	[0.810–0.865]	0.821	[0.796–0.845]	0.799	[0.774–0.823]	4.7%
D2	0.816	[0.793–0.840]	0.791	[0.751–0.830]	0.761	[0.723–0.799]	6.8%
D3	0.823	[0.801–0.844]	0.802	[0.766–0.837]	0.775	[0.739–0.811]	5.7%

For the smaller lesion size (5 mm), the AUC results were lower than for the 8 mm lesion size, as expected. The AUC increased with the dose but never reached a high level of image quality for all CTs. Indeed, the mean AUC over all CT scanners was only 0.86 at 11 mGy and reached 0.91 for the highest dose level (Fig. 3). The use of a dose level lower than the DRL induced a higher loss of image quality in comparison with the 8-mm lesion size (Table 2). The percentage of AUC reduction when decreasing the dose level from 11 to 7 mGy, varied from 3.6 to 6.8% for the various CTs. The AUC ranged from 0.76 to 0.86 at 7 mGy. An AUC of 0.85 was obtained at a range of doses between 6.0 and 14.3 mGy.

### High-contrast detectability

**Fig. 4:**
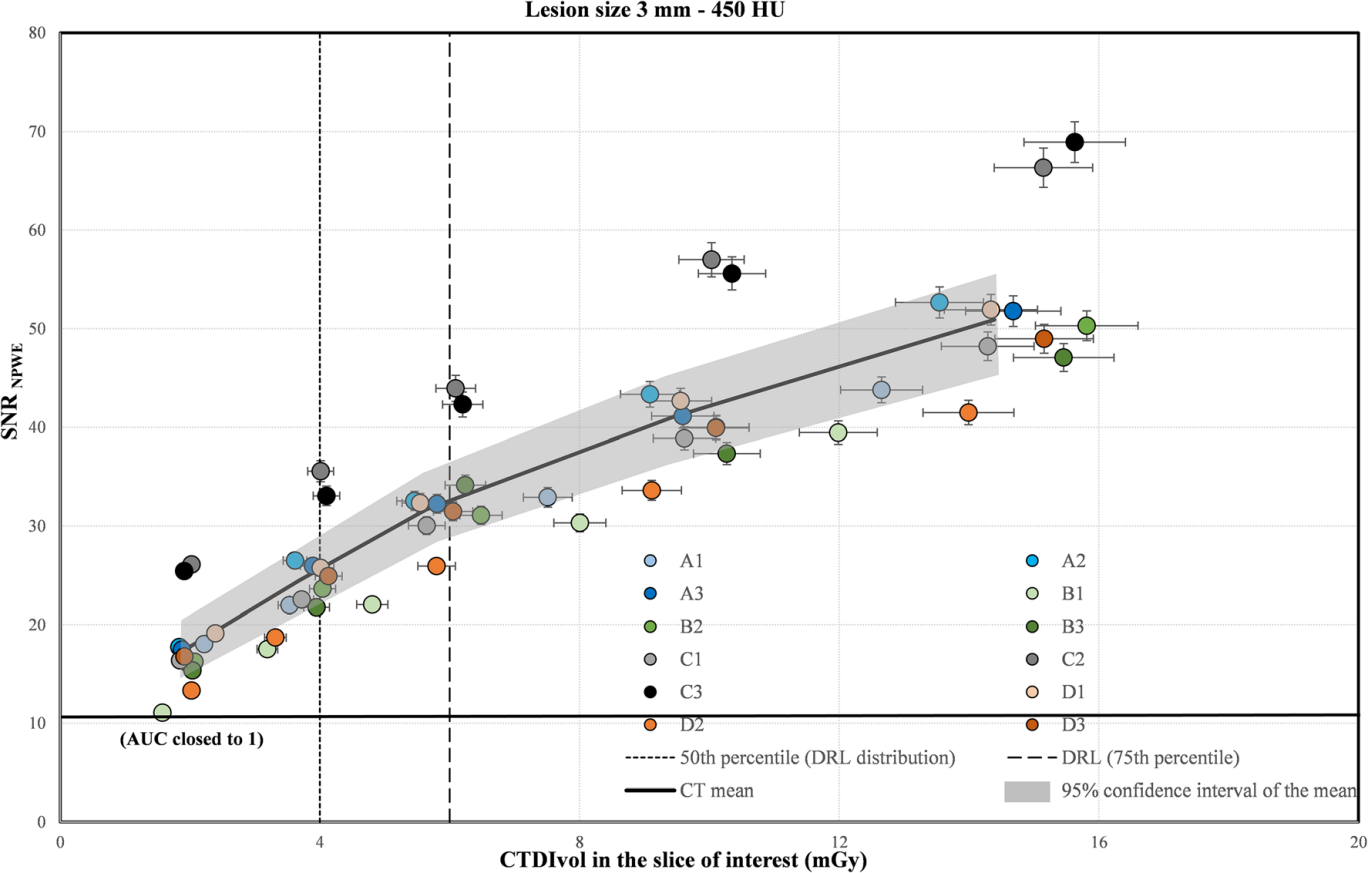
Signal-to-noise ratio (SNR) calculated for the 3-mm lesion size using the NPWE model observer as a function of CTDI_vol_ in the slice of interest for the 12 CT scanners. The vertical and horizontal uncertainty bars represent the expanded uncertainty (k = 2, 95% level of confidence) for the SNR and the CTDI_vol_, respectively. The solid black line was plotted by joining 5 points representing the mean SNR and the mean CTDI_vol_ over all 12 CTs for each dose level. The gray band was plotted by joining the limits of the 95% confidence intervals of the 5 points

**Fig. 5:**
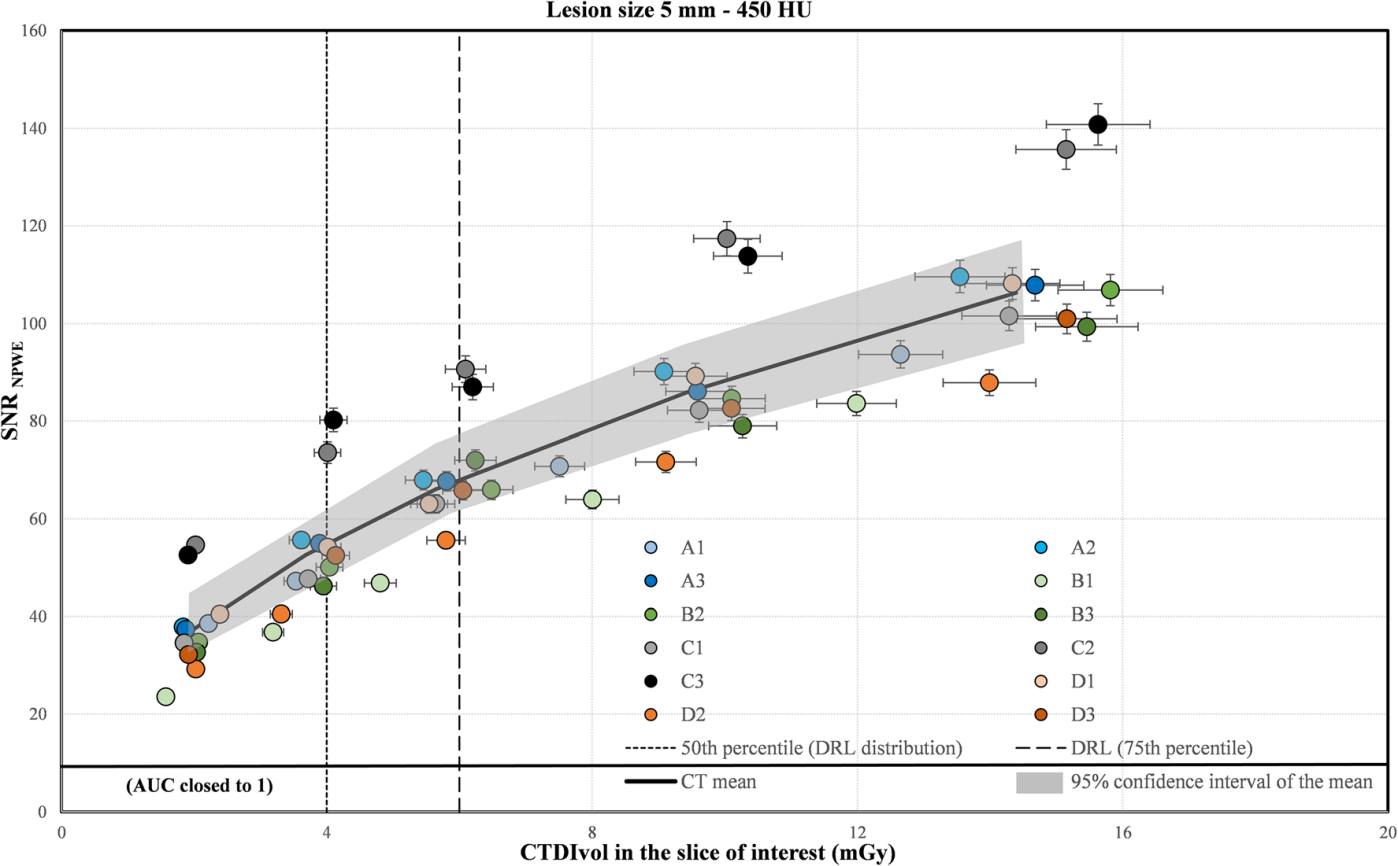
Signal-to-noise ratio (SNR) calculated for the 5 mm lesion size using the NPWE model observer as a function of CTDI_vol_ in the slice of interest for the 12 CT scanners. The vertical and horizontal uncertainty bars represent the expanded uncertainty (k = 2, 95% level of confidence) for the SNR and the CTDI_vol_, respectively. The solid black line was plotted by joining 5 points representing the mean SNR and the mean CTDI_vol_ over all 12 CTs for each dose level. The gray band was plotted by joining the limits of the 95% confidence intervals of the 5 points

The most challenging high contrast task was the detection of a 3 mm calcic lesion (Fig. 4). The results for the 5-mm lesion are presented in Fig. 5. For each CT, the detectability increased with the dose. But even at the lowest dose level (2 mGy), for both lesion sizes, the SNR for all CTs was very high (AUC close to 1.0), indicating that the detection of lesions with such sizes and nominal contrast relative to a homogeneous background was trivial.

## Discussion

In the framework of patient radiation dose optimization, it is essential to ensure that both the dose and image quality are equally balanced to fulfill the diagnostic requirements at the lowest possible dose [13]. The detection of low-contrast lesions in a uniform background is a simple task in comparison with the complexity of a radiological diagnosis for the detection of focal liver lesions. However, even in this simple condition, the task is challenging (Figs. 2 and 3). For the largest lesion size investigated (8 mm), the dose optimization curve reaches a high level of image quality (mean AUC over all CTs higher than 0.95) at approximately 11 mGy (corresponding to the DRL). However, there is a loss of low-contrast detectability for all CTs when using lower dose levels. Our results indicate that one has to be cautious when using doses below the current Swiss DRL (11 mGy) and even more below the 25^th^ percentile (7 mGy), as discussed in ICRP 135 [12]. For the 5-mm lesion size, the task is even more challenging. The detectability never reached a high level of image quality when increasing the dose from 4 to 20 mGy. Furthermore, the variations in image quality between CT scanners should imply a difference of diagnostic information contained in clinical images. Conversely, different doses should be used to achieve the same outcome when dealing with low contrast detection (see Table 2). This shows the limitation of the DRL concept for optimizing radiation dose without assessing image quality. The high contrast detection task was chosen to simulate the detection of renal stones. It appears that this task in homogeneous background is not challenging enough to assess the potential dose optimization. Even for the smallest dose level investigated (2 mGy) and the smallest lesion size (3 mm in diameter), the detectability is very high for all CTs, indicating a perfect detection in this simple condition. Nevertheless, differences in the SNR between the CT scanners were observed for all five dose levels (Fig. 4). With these results, it seems reasonable to hypothesize that correct optimization would lead to different doses on different CT scanners for a more realistic, more challenging high-contrast detection task with anatomical background, or for size, shape, and CT number determination.

The results show that it is necessary to link national DRLs for specific clinical tasks with task-based image quality criteria. In the future, an image quality reference level associated with the DRL could be used for specific clinical tasks [39]. A discussion among the radiologists, the community should also be initiated to define a minimum level of image quality required, depending on the clinical indications, for a safe diagnosis. This could avoid excessive patient dose reduction, in particular for the detection of subtle lesions, as reported by several authors in phantoms [40, 41] and also in patient studies [42]. This approach follows ICRP publication 135, claiming that the “application of DRL values is not sufficient for optimization of protection. Image quality must be evaluated as well” [12]. The assessment of task-based image quality using mathematical observers is an objective and quantitative approach [17] and the outcomes are linked with human observer performances [26, 39]. The phantom presents some limitations. Firstly, the contrast of the various lesions in the phantom was created using plastic materials of low atomic numbers and cannot perfectly simulate the contrast of lesions in a CT acquisition that uses a contrast agent. Ideally, a phantom with iodine lesions should be used to optimize arterial and venous phases of abdominal protocols. Secondly, the background was homogeneous. We should expect that the use of a realistic anatomical background would be more challenging and the AUC results would be worse [43]. CT scanner–specific settings and properties like collimation, flying focus technique, pitch, tube voltage, rotation time, ATCM settings, reconstruction algorithms, slice thickness, and increment are not identical. However, these differences cannot be avoided. Particularly, the 3-mm slice thickness with an increment of 1.5 mm is not optimal to minimize the partial volume effect of the 5 mm lesion size [44]. Moreover, we did not reposition the phantom between scans, so the effect was not averaged out. Furthermore, the standard IEC CTDI_w_ measurement method that was used in this study is known to underestimate CTDI_w_ for wide CT beams because the scatter equilibrium is not achieved [45, 46]. However, no correction factor was applied to the IEC measurements because the collimation was smaller than 40 mm for 11 out of 12 CTs [47]. The described differences in CT scanner specific settings and properties do not allow a completely fair comparison between scanners. However, the goal was not to rate the CT scanners but to study typical CT scanner variability of the image quality at a given dose. Despite the stated limitations, the results show the limitation of the DRL concept. Hence, CT scanner model–specific DRLs could be an option to avoid an unjustified wide dispersion of image quality for well-defined clinical tasks. However, due to the great diversity of CT models and manufacturers on the market, their implementation in clinical routine is difficult. The application of local DRLs to check the clinical practice may be easier to implement using Dose Archiving and Communication Systems (DACS). Ideally, dose optimization should encompass both the DRL process and image quality evaluation using a task-based paradigm. However, the highest priority for the optimization process is to ensure that the image quality is sufficient for the clinical question.

In conclusion, task-based image quality was assessed for various dose levels related to the current DRL values. Assessing image quality metrics related to the clinical question to be answered must be an important part of the optimization process. Comparable image quality for specific clinical questions cannot be reached at the same dose level on all CT scanners. This variability between CTs implies the need for a CT model–specific dose optimization.

## Abbreviations

AUC: Area under the ROC curve
CHO: Channelized Hotelling observer
CTDI_vol_: Volume computed tomography dose index
CTDI_w_: Weighted computed tomography dose index
DDoG: Dense difference of Gaussians
DRL: Diagnostic reference level
HU: Hounsfield units
IR: Iterative reconstruction
NPS: Noise power spectrum
NPWE: Non-prewhitening observer with an eye filter
ROI: Region of interest
SNR: Signal-to-noise ratio
TTF: Target transfer function

## Declarations

### Guarantor

The scientific guarantor of this publication is Dr. Damien Racine.

### Statistics and biometry

One of the authors has significant statistical expertise.

### Methodology

• experimental

• multicenter study
